# Muscle-tendon unit design and tuning for power enhancement, power attenuation, and reduction of metabolic cost

**DOI:** 10.1016/j.jbiomech.2023.111585

**Published:** 2023-04-13

**Authors:** N.C. Holt, D.L. Mayfield

**Affiliations:** Department of Evolution, Ecology and Organismal Biology, University of California Riverside, 900 University Avenue, Riverside, CA 92521, USA

**Keywords:** Muscle–tendon unit morphology, Elasticity, Locomotion, Muscle energetics, Power amplification

## Abstract

The contractile elements in skeletal muscle fibers operate in series with elastic elements, tendons and potentially aponeuroses, in muscle–tendon units (MTUs). Elastic strain energy (ESE), arising from either work done by muscle fibers or the energy of the body, can be stored in these series elastic elements (SEEs). MTUs vary considerably in their design in terms of the relative lengths and stiffnesses of the muscle fibers and SEEs, and the force and work generating capacities of the muscle fibers. However, within an MTU it is thought that contractile and series elastic elements can be matched or tuned to maximize ESE storage. The use of ESE is thought to improve locomotor performance by enhancing contractile element power during activities such as jumping, attenuating contractile element power during activities such as landing, and reducing the metabolic cost of movement during steady-state activities such as walking and running. The effectiveness of MTUs in these potential roles is contingent on factors such as the source of mechanical energy, the control of the flow of energy, and characteristics of SEE recoil. Hence, we suggest that MTUs specialized for ESE storage may vary considerably in the structural, mechanical, and physiological properties of their components depending on their functional role and required versatility.

## 1. Introduction

In skeletal muscle, interactions between contractile proteins consume metabolic energy and generate the force, work, and power required for movement. Regular arrangements of these contractile proteins, sarcomeres, are organized into muscle fibers. It has long been recognized that these muscle fibers operate alongside connective tissue structures, tendons and aponeuroses, in muscle–tendon units (MTUs) (Stensen, 1667; Elliott, 1965). Materials testing of these connective tissue structures has demonstrated that they are elastic, and can stretch (Benedict, 1968; Rack and Westbury, 1984) and recoil, storing and returning elastic strain energy (ESE) (Ker, 1981; Bennett et al., 1986; Pollock and Shadwick, 1994a; Azizi et al., 2009). Analysis of limb and joint mechanics in a jumping dog (Alexander, 1974) and pacing camels (Alexander et al., 1982), recording of muscle spindle afferents in anesthetized cats (Rack and Westbury, 1984), and direct measurements of muscle fiber length in a walking cat (Griffiths 1991) showed that this stretch and recoil of elastic elements also occurred *in viv*o, and could decouple joint rotation and changes in the energy of the body from muscle fiber length changes. The implications of this decoupling for organismal performance were quickly recognized with proposals that tendon elasticity could limit proprioceptive feedback (Rack and Westbury, 1984) and positional control of joints (Pollock and Shadwick, 1994b). While others argued that the interaction of contractile and elastic elements may improve locomotor performance by enhancing (Hill, 1950; Bobbert et al., 1986; Marsh and John-Alder, 1994; Peplowski and Marsh, 1997; Aerts, 1998) and attenuating (Griffith, 1991; Reeves and Narici, 2003; Roberts and Azizi, 2010; Konow et al., 2012) contractile element velocity and power, and reducing metabolic costs (Cavagna et al., 1964; Alexander and Vernon, 1975; Heglund et al., 1982; Alexander and Bennet-Clark, 1977).

**Fig. 1.**
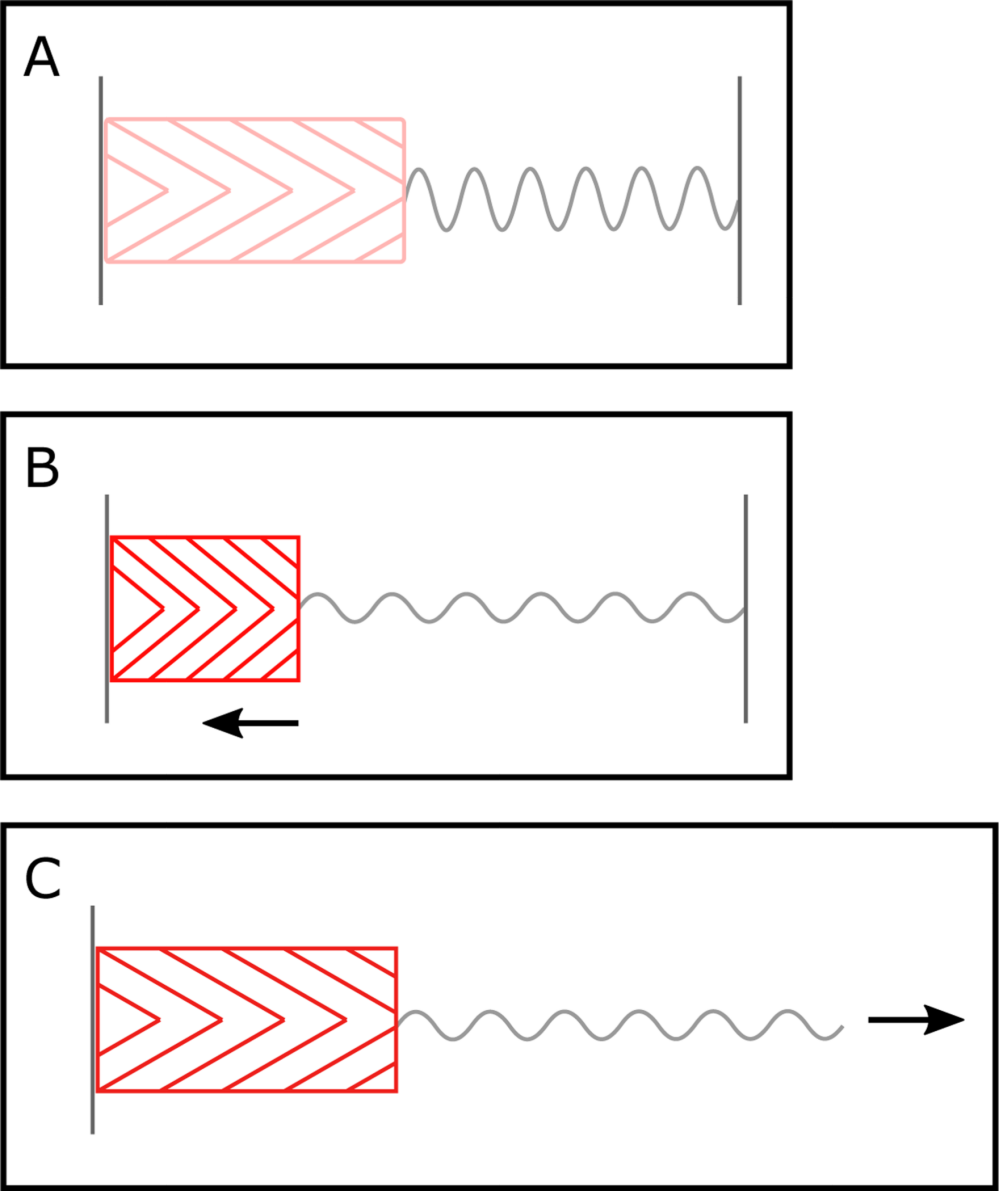
Schematic representations of energy storage in MTUs. A – inactive pennate muscle fibers (pink) are shown organized in series with an elastic element (grey) with both ends of the MTU fixed in place. B – muscle fibers are activated (red) and generate force (black arrow). Both ends of the MTU are fixed in place and the active muscle fibers shorten and stretch the series elastic element. In this case, stored ESE arises from muscle work. C – muscle fibers are activated (red) and the whole MTU is stretched by an external load (black arrow). The active force generated by muscle fibers is sufficient to resist the external force, however, the stiffness of the tendon is not. The tendon is stretched and stored ESE arises from the external work done. (For interpretation of the references to colour in this figure legend, the reader is referred to the web version of this article.)

Considerable variation has been observed in the structural and mechanical properties of contractile and series elastic elements (SEEs) across MTUs (Hill, 1950; Zajac, 1989; Pollock and Shadwick, 1994b; Loren and Lieber, 1995; Ettema, 1996; Biewener and Roberts, 2000; Kargo and Rome, 2002; Ward et al., 2005; Williams et al., 2007; Wilson and Lichtwark, 2011), and in how these components interact to store ESE (Anderson and Pandy, 1993; Roberts and Azizi, 2011; Fig. 1). SEEs can be stretched by muscle fiber shortening (i.e., muscle fibers do work directly on SEEs; Azizi and Roberts, 2010; Fig. 1B), or upon landing and braking with ESE arising from the energy of the body (i.e., external work is done on the MTU; Rack and Westbury, 1984; Roberts et al., 1997; Fukunaga et al., 2001; Fig. 1C).

In this review, we explore the variation in the contractile and elastic components of the MTU in relation to the diversity of potential locomotor benefits of ESE storage in terrestrial vertebrates. We review historical and contemporary literature from human and comparative fields on: 1) the structural and mechanical properties of the contractile and elastic components of MTUs; 2) the design and tuning of MTUs for ESE storage; 3) the benefits of ESE storage for locomotor performance; and 4) the potential for MTUs to be specialized for enhancing specific features of locomotor performance.

## 2. Muscle-tendon units

Muscle-tendon units contain contractile and elastic elements. Actin (Hanson and Lowy 1963), myosin (Huxley 1963), and potentially the molecular spring titin (Maruyama, 1976), are the contractile components of MTUs (Huxley and Niedergerke, 1954; Huxley and Hanson, 1954; Kellermayer and Granzier, 1996). These contractile proteins are organized into sarcomeres which are arranged, in series and parallel, into muscle fibers. Muscle fibers are arranged either parallel, or at some angle (the pennation angle), to the line of action of the muscle (Gans, 1982; Otten, 1988; Wilson and Lichtwark, 2011), and are surrounded by the extracellular matrix (ECM) (Borg and Caulfield, 1980; Purslow and Trotter, 1994; Kjaer, 2004; Sleboda et al., 2020). The ECM inserts onto tendinous sheets, or aponeuroses (Lieber et al., 1991; Scott and Loeb, 1995; Azizi et al., 2009), which narrow to tendons (Elliott, 1965; Alexander, 2002). Titin, ECM, aponeuroses, and tendons all have some degree of elasticity (Roberts, 2016; Williams and Holt, 2018; Holt, 2019), with tendons and aponeuroses considered to be the primary elastic elements in MTUs (Alexander and Bennet-Clark, 1977; Huijing, 1991; Alexander, 2002; Roberts, 2016). Titin and the ECM are generally considered to be in parallel with contractile elements, and to define the passive stiffness of the muscle fibers (Meyer and Lieber, 2011, 2018). Tendons are in series with muscle fibers (Hill, 1950); they are stretched by active muscle fiber shortening (Fig. 1B) and by externally applied loads (Fig. 1C). Aponeuroses can also stretch upon the development of muscle force (Maganaris and Paul, 2000; Roberts et al., 1997; Azizi and Roberts, 2009). However, their relationship between force and length is more complex than for tendon (Zuurbier et al., 1994; Raiteri et al., 2018; Herzog, 2019; Bossuyt et al., 2023).

### 2.1. Structure, function, and diversity in contractile elements

Upon muscle activation myosin heads can bind to actin, cyclically forming crossbridges, and titin stiffness increases (Kellermayer and Granzier, 1996; Labeit et al., 2003; Nishikawa et al. 2012; Dutta et al., 2018). Both these changes increase muscle fiber stiffness (Labeit et al., 2003; Powers et al., 2014). However, crossbridge cycling also acts to shorten the sarcomere and muscle fiber (Huxley 1953; Huxley and Hanson 1954; Huxley and Niedergerke 1954; Huxley 1957, 1969). The length change that muscle fibers undergo while producing force is key to their mechanical function. External forces lower than, equal to, or greater than muscle force will result in shortening contractions, isometric contractions, and lengthening contractions, respectively (Griffiths, 1991; Roberts et al., 1997; Biewener et al., 1998; Fukunaga et al., 2001; Carr et al., 2011). Metabolic energy is consumed as adenosine triphosphate (ATP) is hydrolysed by myosin heads during crossbridge cycling (Engelhardt and Ljubimowa 1939; He et al., 2000).

The capacity of the contractile elements to generate force, do work, produce power, and absorb energy depends on contractile conditions under which they operate, and muscle morphology and physiology. In a given muscle, the active force that can be produced is highly dependent on sarcomere length and rate of length change, as described by the force–length and force–velocity relationships. Isometric force is maximal at an intermediate sarcomere length corresponding to optimal actin-myosin overlap (Gordon et al., 1966; Herzog et al., 1992). Muscle force declines with increasing shortening speed (Hill, 1938; Alcazar et al., 2019), and increases, up to a point, with increasing lengthening speed (Abbott et al 1952; Nishikawa 2016). When muscle fibers shorten, they do mechanical work equal to the dot product of their force and displacement vectors, and generate instantaneous mechanical power equal to the dot product of their force and velocity vectors. Work and power are limited by force–length and force–velocity relationships, with work being maximized by slow length changes across the plateau of the force–length relationship (Peplowski and Marsh, 1997; Azizi and Roberts, 2010) and power being maximized at intermediate velocities (Barclay et al., 1993; Bottinelli et al., 1996).

Across MTUs, force can be increased by increasing the number of sarcomeres in parallel, or physiological cross-sectional area (XSA_phys_), often by increasing pennation angle (Powell et al., 1984; Wilson and Lichtwark 2011). The capacity of a muscle to change length without sacrificing significant force due to force–length and force–velocity effects, so enhancing work and power, can be increased by increasing the number of sarcomeres in series, or muscle fiber length (Bodine et al., 1982; Powell et al 1984; Wilson and Lichtwark, 2011). Maximum shortening velocity and power can be increased further by increasing the proportion of faster muscle fiber types containing faster myosin isoforms (Rome et al., 1988; Bottinelli et al., 1996; Schiaffino and Reggiani, 2011).

Contractile conditions and muscle morphology and physiology affect not only muscle force, work, and power, but also the metabolic cost per unit force and efficiency. The cost per unit force is higher during shortening than isometric contractions, due to an increased rate of crossbridge cycling (Fenn, 1923; Rall, 1982; Barclay, 2022) and force–velocity effects (Roberts et al., 1997; Fletcher and MacIntosh, 2017; Bohm et al., 2019), and lower during lengthening contractions (Abbott et al., 1952; Curtin and Davies, 1973; Beltman et al., 2004; Holt et al., 2014), possibly due to the increased force contributions of titin (Nishikawa, 2016). Muscle efficiency varies with muscle shortening velocity and is typically maximal at ~ 20% of maximum shortening velocity (Barclay et al., 1993). However, muscle energetics may be complicated by the activation state of the muscle (Lou et al., 1998) and its contractile history (Joumaa and Herzog, 2013; Holt et al., 2014; Curtin et al., 2019).

Across different MTUs, energy consumption is determined by the rate of crossbridge cycling and force generation (Barclay et al., 1993; Roberts et al., 1998a; He et al., 2000; Beck et al., 2020), and the volume of muscle active (Alexander, 1991; Roberts et al., 1998b; Biewener and Roberts, 2000). MTUs with larger contractile elements and faster muscle fiber types consume more metabolic energy. However, variation in metabolic energy consumption is best considered alongside variation in mechanical output. For example, the dependency of force on XSA_phys_, and metabolic cost on volume, means that reduced muscle fiber length will decrease the cost per unit of force. And faster contractile elements will increase the cost of force production, but may allow relatively high efficiencies to be sustained at higher absolute shortening velocities (Barclay et al., 1993).

### 2.2. Structure, function, and diversity in elastic elements

Tendons and aponeurosis are the primary elastic elements, potentially in series with contractile elements, in MTUs. Tendons are composed of highly-organised collagen fibrils aligned parallel to the long axis of the tendon (Elliott, 1965; Jozsa et al., 1991). They deform in a characteristic and somewhat reproducible manner when loaded both by external forces and muscle fibers (Bennett et al., 1986; Pollock and Shadwick, 1994a; Cui et al 2009), indicating a mechanically in-series operation with contractile elements (Herzog, 2019). Tendon loading gives a J-shaped stress–strain response whereby modulus is initially low, representing a compliant “toe” region, and gradually increases to a constant, higher value at large loads (Bennett et al., 1986; Pollock and Shadwick, 1994a). Tendon hysteresis is relatively low, with <10% of the energy stored typically lost on recoil (Bennett et al., 1986; Shadwick, 1990; Pollock and Shadwick, 1994b; Wilson and Goodship, 1994).

Aponeuroses can constitute a significant proportion of total tendinous tissue length within an MTU (Lieber et al., 1991; Trestik and Lieber, 1993; Zuurbier et al., 1994; Maganaris and Paul, 2000). They have a similar pattern of collagen orientation to tendons, a superficially similar stress–strain response (Lieber et al., 1991; Zuurbier et al., 1994; Scott and Loeb, 1995; Maganaris and Paul, 2000; Muramatsu et al., 2001; Monti et al., 2003; Stafilidis et al., 2005), and have been reported to undergo similar (Trestik and Lieber, 1993; Scott and Loeb, 1995; Muramatsu et al., 2001), or greater (Huijing and Ettema 1988; Lieber et al 1991; Maganaris and Paul, 2000; Monti et al., 2003) strains. Hence, aponeuroses could be important sites of ESE storage within MTUs. However, significantly lower strain in aponeuroses than tendons (Magnusson et al., 2003; Iwanuma et al., 2011), and considerable variation in strain along their length (Zuurbier et al., 1994; Maganaris and Paul, 2000), have also been reported. More importantly, aponeuroses may not exhibit characteristic and reproducible extensions in response to force, and so may not function as simple in-series elastic elements (Ettema and Huijing, 1989; Zuurbier et al., 1994; Scott and Loeb, 1995; Lieber et al. 2000; Azizi and Roberts, 2009; Arellano et al., 2016; Raiteri et al., 2018; Arellano et al 2019; Herzog, 2019; Bossuyt et al., 2023). For example, the turkey lateral gastrocnemius aponeurosis is 50% stiffer when loaded actively compared to passively (Azizi and Roberts, 2009), likely due to the width-wise expansion that results from muscle fiber bulging during active shortening (Zuurbier et al., 1994; Scott and Loeb, 1995; van Donkelaar et al., 1999; Azizi and Roberts, 2009; Iwanuma et al., 2011; Raiteri et al., 2016; Raiteri et al., 2018; Roberts, 2019). This variable stiffness is likely to affect aponeurosis capacity for ESE storage and return (Arellano et al., 2019; Herzog, 2019), and it has been suggested that aponeuroses function simply to transmit force, while the tendon stores ESE (Zuurbier et al., 1994; Magnusson et al., 2003). However, ESE storage and return has been clearly demonstrated in MTUs in which the majority of the elasticity resides in the aponeurosis rather than the tendon (Roberts et al., 1997; Konow et al., 2012). Hence, while aponeuroses can, at least in some cases, store and return ESE, the mechanics of this remain poorly understood.

SEE stiffness, determined by modulus and geometry, is a major determinant of ESE storage capacity (Alexander and Bennet-Clark, 1977; Fukashiro et al., 1995). Across many mammalian species and muscles, the stress–strain relationship of tendon, and therefore its modulus, is largely invariant. The linear region of this relationship is attained at stresses of approximately 25–30 MPa, and has a modulus of 1.24–1.5 GPa (Bennett et al., 1986; Pollock and Shadwick, 1994b). Subsequent studies have showed some variation in modulus across functionally diverse muscles (Shadwick, 1990; Matson et al., 2012; Javidi et al., 2019), and with inactivity (Almeida-Silveira et al., 2000; Reeves et al., 2005; Couppé et al., 2012). However, it appears that a consistent modulus may be attained in all tendons with sufficient, long-term, loading (Woo et al., 1980, 1981; Buchanan and Marsh, 2001; Seynnes et al., 2009; Heinemeier et al., 2012; Geremia et al., 2018; Massey et al., 2018a; Massey et al., 2018b; Waugh et al., 2018; Centner et al., 2019; Charcharis et al., 2019; Walker et al., 2020; Quinlan et al., 2021). In contrast to tendon, there are relatively few measurements of aponeurosis modulus. Studies on isolated aponeuroses from turkey and human gastrocnemius muscles report values of 0.75 (Azizi and Roberts, 2009) and 0.2–0.3 GPa (Shan et al., 2019), respectively. However, no extensive comparative studies exist.

Despite their relatively consistent material properties, tendons can vary broadly in their stiffness as a result of variation in their geometry and operating stresses; increased length, reduced XSA, and reduced operating stress are all known to reduce the effective stiffness of tendon (Zajac, 1989; Hoy et al., 1990; Pollock and Shadwick, 1994b; Loren and Lieber, 1995; Ker et al., 1988 Kargo and Rome, 2002). Similar variability presumably exists in aponeuroses, although, as with modulus, aponeurosis morphology and effective stiffness are relatively unstudied in a comparative context. In order to understand the functional consequences of variation in tendon and aponeurosis properties, they must be considered in the context of variation in contractile tissue.

## 3. Design and tuning of muscle–tendon units

The relationship between contractile and elastic elements in MTUs has been considered in two major ways. Historically, variation in MTU design has been described based on the structural and functional properties of the contractile and elastic elements. More recently, the relationship between contractile and series elastic elements has been described in terms of “tuning” of the MTU (Galantis and Woledge, 2003; Lichtwark and Wilson, 2008; Fletcher et al., 2010; Ilton et al., 2018; Mendoza and Azizi, 2021).

### 3.1. MTU design

MTU structural design is often described by the ratio of tendon to muscle fiber length. This ratio tends to increase distally across the limb and varies considerably in homologous muscles across species (Hoy et al., 1990; Zajac, 1989; Williams et al., 2007; Ettema, 1996). Human gluteal, quadriceps, and triceps surae muscle groups have tendon-to-fiber length ratios of ~ 0.25, ~4, and ~ 10, respectively (Hoy et al., 1990), and values of 2.2, 3.7, and 7.0 have been reported for the gastrocnemius muscles of rats, hopping mice, and wallabies respectively (Ettema, 1996). Relatively longer tendons will decrease tendon stiffness and increase ESE storage for a given load (Ettema, 1996; Hoy et al., 1990; Kargo and Rome, 2002; Loren and Lieber, 1995; Pollock and Shadwick, 1994b; Zajac, 1989). However, relatively short muscle fibers may not be able to perform sufficient work on a long compliant tendon. In which case, external work may be required to store significant ESE (Ker et al., 1988; Pollock and Shadwick, 1994b).

MTU functional design has been described by tendon operating stresses, fiber length factors, and fixed-end compliance. Maximal tendon operating stresses have been directly measured (Lieber et al., 1991; Maganaris and Paul, 2000; Reeves et al., 2003; Cui et al., 2009; Stenroth et al., 2012) or estimated from measurements of muscle and tendon XSA (Ker et al., 1988; Pollock and Shadwick, 1994b; Wilson and Lichtwark, 2011), and compared to measured or assumed tendon stress–strain curves to determine potential strain, operating region on the stress–strain curve, and ESE storage. MTUs specialized for ESE are assumed to operate in the linear region of their stress–strain curves (Pollock and Shadwick, 1994b), and operating stress typically varies with body size due to the presence of relatively thicker tendons in smaller species (Pollock and Shadwick, 1994b). For example, estimated maximum operating stress for the gastrocnemius tendon in the hopping mouse is 19 MPa, compared to 62 MPa in the red-bellied Pademelon (Ettema, 1996).

Fiber length factors and fixed-end compliance expand on operating stresses by estimating or measuring tendon elongation, and expressing this relative to muscle fiber length (Ker et al, 1988; Griffiths, 1991; Pollock and Shadwick, 1994b; Williams et al., 2007; Wilson and Lichtwark, 2011, Biewener and Roberts, 2000). Fiber length factors use predictions of muscle force and tendon stiffness to estimate tendon deformation, which is then normalized to muscle fiber length (Pollock and Shadwick, 1994b). These metrics have been used to estimate the degree of specialization of an MTU for ESE, and ability of the muscle to stretch the tendon and so control joint position. Assuming a maximum physiologically realistic muscle fiber shortening of 25% (Dimery, 1985; Ker et al., 1988), fiber length factors < 4 were interpreted as muscle fibers no longer being able to fully stretch the tendon and so unable to control joint position, and fiber length factors of < 2 were assumed to indicate complete specialization for ESE storage rather than joint positioning (Pollock and Shadwick, 1994b).

Fixed-end compliance has been determined by direct measurement of muscle fiber shortening against series elasticity, normalized to muscle fiber length (Griffiths, 1991; Scott and Loeb, 1995; Kawakami and Lieber, 2000; Buchanan and Marsh, 2001; Roberts, 2002; Karamanidis et al., 2005; Sawicki et al., 2015; Ahn et al., 2018; Moo et al., 2020; Mendoza and Azizi, 2021). This direct measurement is beneficial as it accounts for all elasticity, including the often ignored but likely important aponeurosis, and incorporates the effects of force–length and force–velocity relationships on the capacity of contractile elements to generate force and stretch SEEs. Values ranging from ~ 10% in the mouse tibialis anterior (Moo et al., 2020) to ~ 28% in the rat gastrocnemius (Ahn et al., 2018), and ~ 45% in the the Cuban tree frog (Mendoza and Azizi, 2021) have been reported. It should be noted that only the latter MTU even approaches a fiber length factor value suggested to indicate specialization for ESE storage according to this metric (1/0.45 = 2.22; Ker et al., 1988; Pollock and Shadwick 1994b).

### 3.2. MTU tuning

**Fig. 2.**
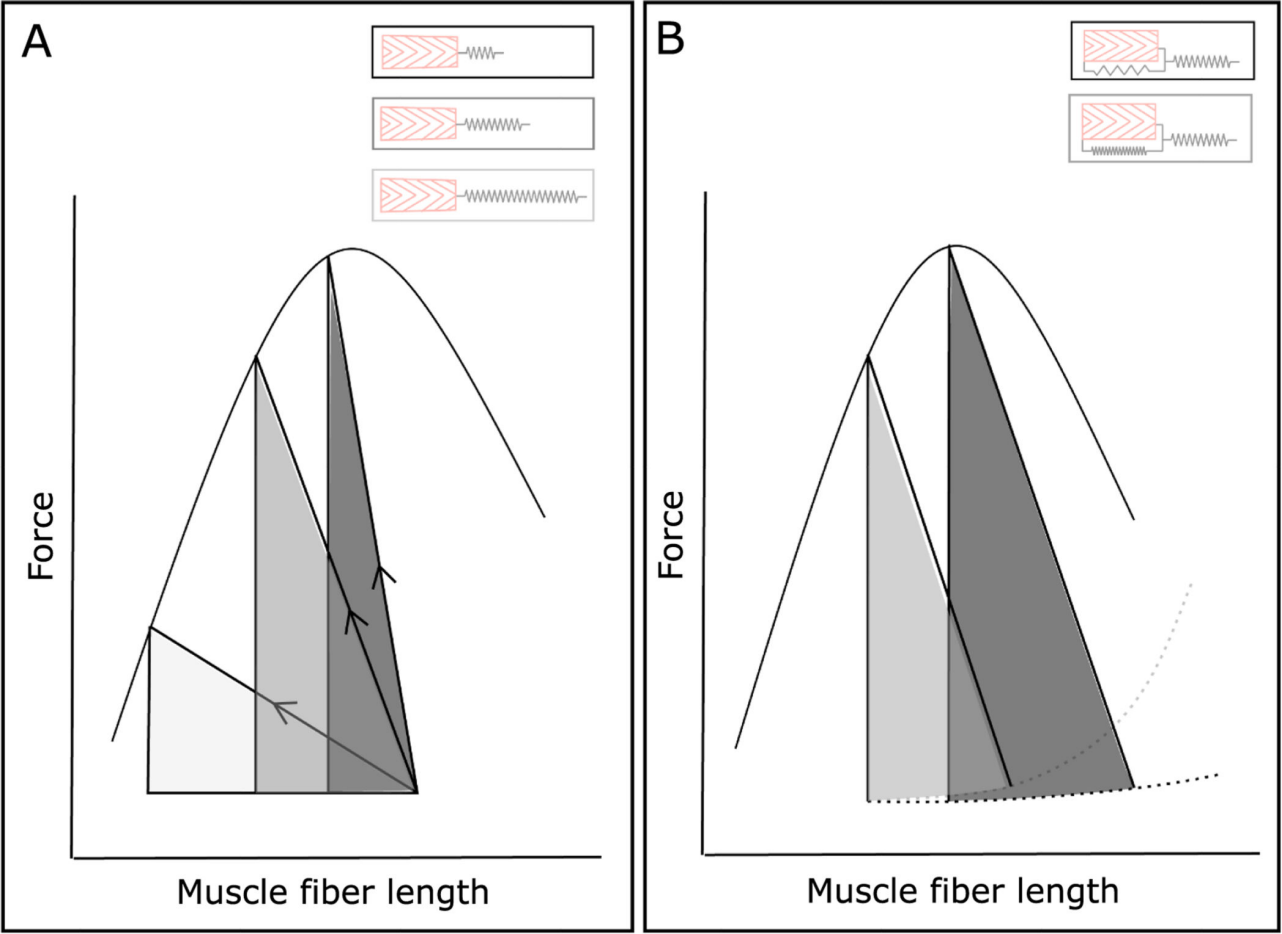
Schematic representation of the effects of MTU tuning on ESE storage. Muscle active and passive force–length curves are shown in solid lines and dashed lines respectively. Upon muscle activation, muscle fibers shorten along the trajectory indicated by the arrows with the slope of the line determined by the stiffness of the SEE. The shaded triangles indicate the energy stored in the SEE by muscle fiber shortening during a fixed-end contraction. A - The effect of SEE stiffness on muscle fiber shortening and ESE storage. Fibers shorten (and so SEEs stretch) most with a low stiffness (light grey), and least with a high (dark grey) stiffness. The most ESE is stored with intermediate stiffness (mid-grey). B – The effect of muscle passive stiffness and muscle fiber operating lengths on ESE. Lower passive muscle stiffness (dark grey) allows for increased muscle fiber length at the start of the contraction and so increases ESE storage compared to the stiffer muscle (light grey). Adapted from Azizi and Roberts, 2010; Cox et al., 2021; Mendoza and Azizi, 2021

In contrast to structural and functional descriptions of MTU design, which provide means of describing differences between MTUs, MTU tuning instead describes the matching of contractile and SEE properties for ESE storage (Fig. 2; Rosario et al., 2016; Ilton et al., 2018; Mendoza and Azizi, 2021). If SEEs are too stiff relative to the force capacity of the contractile element, the SEE cannot be stretched by the muscle, and the contractile element will instead stretch instead of the SEE when the MTU is loaded by external forces. If the SEE is too compliant relative to the force and length-change capacity of the muscle fibers, muscle fibers will undergo significant shortening strains to stretch elastic elements enough to store significant ESE. This will reduce muscle work capacity, due to force–length and force–velocity effects, and so ESE storage (Fig. 2A; Rosario et al., 2016).

Consideration of MTU tuning has also highlighted the potential importance of the passive stiffness of muscle fibers for ESE storage. Passive stiffness is likely to affect the operating range of muscle fibers, with lower passive stiffnesses allowing fibers to be stretched to longer lengths (Fig. 2B). This would allow muscle fibers to shorten over the plateau of the force–length relationship as they stretch elastic elements, and so incur a reduced force penalty compared to muscle starting at the plateau of the force–length relationship (Fig. 2B; Azizi and Roberts, 2010; Cox et al., 2021).

**Fig. 3.**
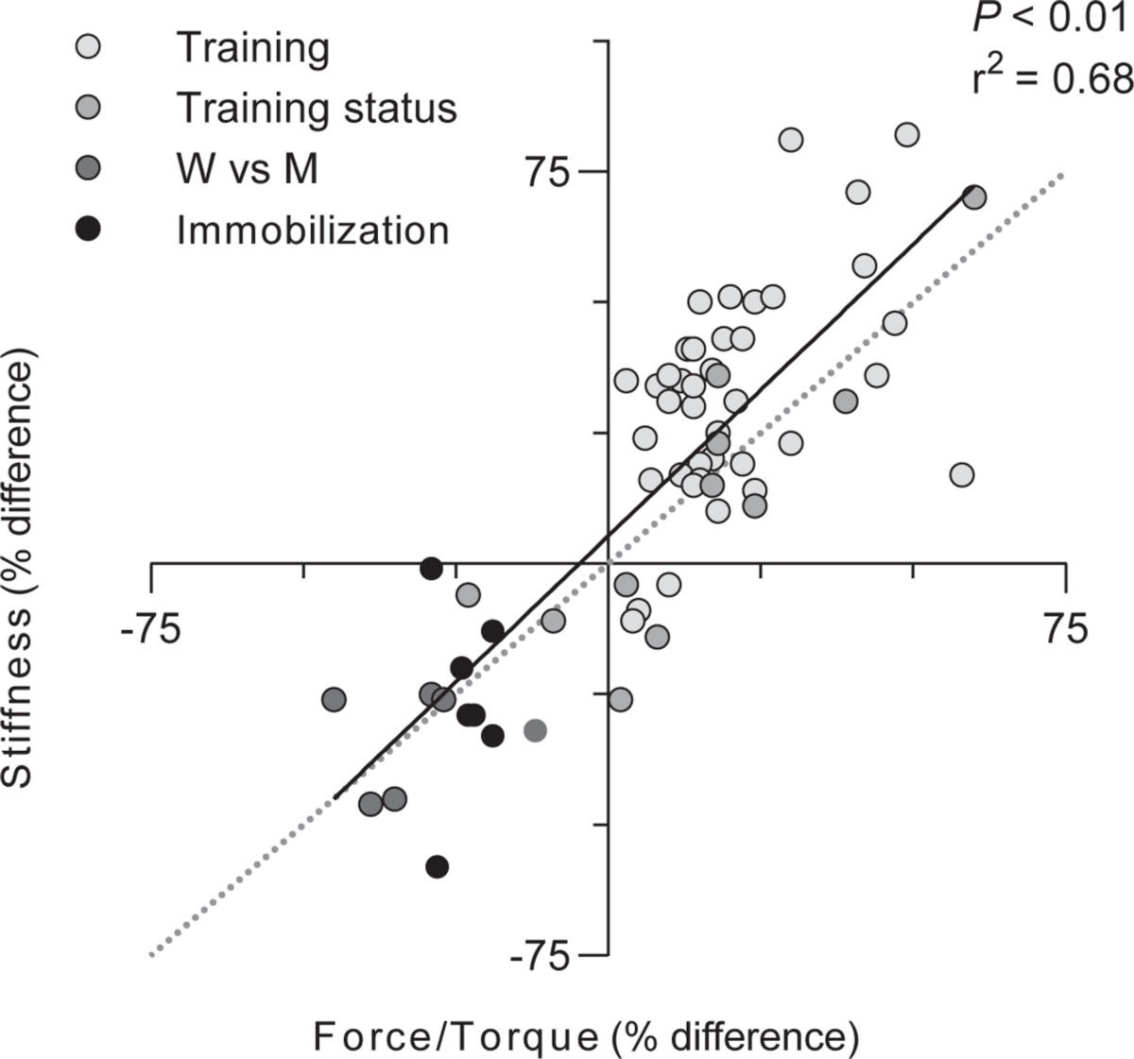
Relationship between changes (i.e., following an intervention) or differences (i.e., between groups) in metrics of muscle strength and changes or differences in tendon or tendinous tissue (tendon and aponeurosis) stiffness. Data are fitted with a linear regression (black line). Dotted line (grey) represents unity. Data values and sources can be found in supplemental table 1. W, women. M, men.

Evidence for the importance of this tuning in MTUs is thought to be provided by the coordinated changes in contractile and SEE properties (Elliott, 1965) across activity levels in an individual (Fig. 3), across individuals in a population (Fig. 3), and across species (Mendoza and Azizi, 2021). Increased loading of MTUs increases muscle strength and tendon stiffness in rough proportion to one another (Arampatzis et al., 2007a; Geremia et al., 2018; Waugh et al., 2018). Differences in Achilles tendon stiffness between men and women, and endurance runners and sprinters, are related to differences in muscle strength (Arampatzis et al., 2007b; Morrison et al., 2015; Muraoka et al., 2005). And Cuban tree frogs show elevated muscle strength and elastic element stiffness compared to cane toads and bullfrogs (Mendoza and Azizi, 2021).

Variation in MTU design and tuning has been suggested to explain variation in ESE storage and locomotor performance. Differences in tendon-to-fiber length ratios have been suggested to explain the 4-fold greater capacity for ESE storage in wallabies compared to rats (Ettema, 1996), and to contribute to the greater jump performance in Cuban tree frogs compared to cane toads (Roberts et al., 2011). Parallel increases in muscle strength and tendon stiffness and have been suggested to improve running economy in humans (Bohm et al., 2021a) and jump performance in frogs (Mendoza and Azizi, 2021). And lower muscle fiber passive stiffness and/ or longer operating lengths have been suggested to have the potential to increase muscle work and ESE storage in jumping frogs (Azizi and Roberts, 2010; Azizi, 2014) and guinea fowl (Cox et al., 2021). Understanding the relationships between contractile and SEEs, both in terms of MTU design and tuning, is clearly key for understanding ESE storage capacity and locomotor performance. However, what becomes apparent from the consideration of various metrics of MTU design, and a new appreciation of MTU tuning, is that optimal MTU properties may vary depending on the source of mechanical energy responsible for ESE in SEEs, and the feature of locomotor performance that is to be enhanced.

## 4. Enhancement of locomotor performance by elastic strain energy storage in series elastic elements

The storage of significant ESE in SEEs has been demonstrated to improve locomotor performance (Roberts and Azizi, 2011) by enhancing (Hill, 1950; Bobbert et al., 1986; Peplowski and Marsh, 1997; Aerts, 1998) and attenuating muscle power (Griffith, 1991; Reeves and Narici, 2003; Konow et al., 2012), and by reducing muscle metabolic energy consumption (Cavagna et al., 1964; Alexander and Vernon, 1975; Roberts et al., 1997).

### 4.1. Power enhancement and amplification

The recoil of SEE following ESE storage allows MTU velocity, and therefore the instantaneous power, to exceed that of the contractile element during MTU shortening. This discrepancy has been termed power enhancement or, in the case where MTU power exceeds maximum contractile element power, power amplification (Richards and Sawicki, 2012). Power enhancement and amplification have been studied extensively in jumping vertebrates (Bobbert et al., 1986; Marsh, 1994; Marsh and John-Alder, 1994; Peplowski and Marsh, 1997; Aerts, 1998; Bobbert, 2001; Kurokawa et al., 2001; Roberts and Marsh, 2003; Henry et al., 2005; Azizi and Roberts, 2010; Astley and Roberts, 2012; Longo et al., 2019; Mendoza and Azizi, 2021). Contractile elements are typically considered do work slowly on SEEs prior to take-off, with a latching mechanism thought to be required to prevent movement of the body during this time (Galantis and Woledge, 2003; Roberts and Marsh, 2003; Astley and Roberts, 2014; Sawicki et al., 2015; Ilton et al., 2018; Longo et al., 2019; Olberding et al., 2019; Roberts, 2019; Divi et al., 2020). SEEs then recoil rapidly during take-off enhancing or amplifying muscle power. This slow loading of SEEs is thought to reduce force–velocity penalties, so increasing contractile element work, while still allowing for rapid take-off as the SEE recoils (Peplowski and Marsh, 1997; Roberts and Marsh, 2003; Robertson et al., 2018; Marsh, 2022). A sevenfold amplification of power has been reported in jumping Cuban tree frogs (Peplowski and Marsh, 1997; Roberts and Marsh, 2003); and MTU power and velocity have been estimated to be 2–3 times that of muscle fibers in jumping humans (Bobbert, 2001; Kurokawa et al., 2001).

In jumping frogs, the plantaris muscle is activated and shortens, so doing work and stretching SEEs, prior to take-off (Azizi and Roberts, 2010; Astley and Roberts, 2012). Latching has been suggested to be achieved through an increasing mechanical advantage at the ankle (Roberts and Marsh, 2003; Astley and Roberts, 2014). This strategy of contractile element work being stored as ESE in SEEs, rather than done directly on the environment, is thought to only be beneficial at small body sizes (Marsh and John-Alder, 1994; Sutton et al., 2019; Mendoza et al., 2020) where take-off time is very limited, as contractile elements can only store a fraction of their total possible work in a SEE (Sutton et al., 2019). However, at least in frog jumping, muscle fibers contribute to shortening throughout take-off (Azizi and Roberts, 2010), and so also do work directly on the environment. This suggests the possibility of an optimal distribution of muscle work stored in SEEs versus done directly on the environment (Robertson et al., 2018), that may vary with body size (Sutton et al., 2019) and jump distance (Marsh, 2022).

Some degree of power amplification has been reported in larger animals including humans, guinea fowl, and dogs (Alexander, 1974; Bobbert, 2001; Kurokawa et al., 2001; Henry et al., 2005), despite the apparent size-based limitations. For humans, 17–50% of jump energy has been shown to arise from elastic recoil (Bosco et al 1982; Bobbert et al 1986; Anderson and Pandy, 1993; Fukashiro et al 1995). However, at least in humans, ESE often comes from the downward movement of the body in the preparatory phase of the jump, rather than directly from contractile element shortening (Anderson and Pandy, 1993; Henry et al., 2005; Farris et al., 2016; Bishop et al., 2021). This energy flow does not require latching, and the MTU is unloaded by the proximal-to-distal extensions of joints across the limb during take-off (Bobbert and van Ingen Schenau, 1988; Pandy and Zajac, 1991; Bobbert, 2001). Hence, while power enhancement has been observed extensively in both jumping frogs and humans, the source of mechanical energy and the control of the flow of energy through the system appear to be fundamentally different, in ways that may be related to body size and degree of specialization for jumping (Alexander, 1995; Sutton et al., 2019; Bishop et al., 2021).

### 4.2. Power attenuation

The storage of ESE in SEEs can reduce the lengthening velocity of contractile elements, and so attenuate negative muscle power, during energy dissipating activities such as landing, descent, or braking. The low hysteresis of SEEs (Bennett et al., 1986; Pollock and Shadwick, 1994a; Wilson and Goodship, 1994) makes them unlikely to dissipate significant energy during such activities (Roberts and Konow, 2013). However, their ability to decouple contractile element length changes from MTU stretch (Griffiths, 1991; Reeves and Narici, 2003; Spanjaard et al 2007; Hoffman et al., 2014; Smith et al., 2022) may improve locomotor performance by minimizing muscle damage (Konow et al., 2012; Roberts and Konow, 2013). During both *in situ* tests and drop landings in turkeys, energy was initially rapidly transferred to the SEE, and then to muscle fibers at a much lower rate. This reduced contractile element lengthening velocity and forces, and so could minimize muscle damage (Roberts and Azizi, 2010; Konow et al., 2012; Roberts and Konow, 2013). SEEs may also allow for safer muscle operating lengths during unexpected perturbations. Muscle fibers in the human distal limb shortened against SEEs prior to ground contact during an unexpected drop perturbation, resulting in shorter muscle fibers that were less likely to be stretched to damaging lengths during energy absorption (Dick et al., 2021).

### 4.3. Reducing metabolic cost of locomotion

The storage of ESE in SEEs has been suggested to reduce the metabolic cost of steady-state locomotion by reducing the need for contractile element work (Cavagna et al., 1968; Alexander, 1991; Voigt et al 1995; Roberts et al., 1998b; Alexander, 1974; Holt et al., 2014; Fletcher and MacIntosh, 2015; Labonte and Holt, 2022). In bouncing gaits such as running and hopping, MTUs must generate force to support bodyweight and accommodate the sequential decrease and increase in kinetic and potential energy that occurs during stance (Cavagna et al., 1964, 1976; Holt et al., 2014). In the absence of SEEs this would be done by active muscle stretch and shortening. However, in MTUs with significant SEEs, contractile elements can generate force isometrically with energy fluctuations accommodated by SEE stretch and recoil. Muscle fibers in distal MTUs often operate quasi-isometrically, doing little work (Biewener and Baudinette, 1995; Roberts et al., 1997; Biewener, 1998; Biewener and Roberts, 2000; Lichtwark et al., 2007; Dean and Kuo, 2011; Eng et al., 2019; Biewener, 1998; McBride, 2021); minimal length changes are seen in the wallaby plantaris (Biewener et al., 1998), and the return of ESE accounts for 16–60% of positive MTU work in hopping humans (Thys et al., 1975; Fukashiro et al 1995; Voigt et al., 1995; Lichtwark and Wilson, 2005; Dean and Kuo, 2011; McBride, 2021). The metabolic benefits of this strategy are supported by the dependence of the metabolic cost of running on force generation (Taylor et al 1980; Kram and Taylor, 1990; Roberts et al., 1998a; Roberts et al., 1998b; Heglund et al., 1982; Beck et al., 2020), the maximization of ESE storage and minimization of metabolic cost at resonant hopping frequencies (Dean and Kuo, 2011; Robertson and Sawicki., 2015), and decreased cost of running with changes to tendon stiffness (Arampatzis et al., 2006; Fletcher et al., 2010).

The metabolic benefit from this avoidance of costly muscle shortening (Fenn, 1923; Holroyd et al., 1996; Roberts et al., 1997; Roberts, 2002; Beltman et al., 2004; Farris and Sawicki, 2012; Fletcher and MacIntosh, 2017; Bohm et al 2019; Barclay, 2022) has however been questioned by the finding that under some, but not all (Van der Zee et al., 2019), conditions, force can be produced as cheaply during active stretch-shorten contractions as during isometric ones (Curtin et al., 2019; Holt et al., 2014). This has been attributed (Holt et al., 2014) to the low cost of force during and following active stretch (Abbott et al., 1952; Beltman et al., 2004; Joumaa and Herzog, 2013), and muscle work being done during relaxation (Lou et al., 1998). This finding requires a more nuanced consideration of muscle energetics, but does not necessarily negate the energetic benefits of SEEs. Reduced need for contractile element work may have allowed for a reduction in muscle volume, which reduces the cost of force and limb inertia (Alexander, 1991; Biewener and Roberts, 2000; Holt et al., 2014; Labonte and Holt, 2022). It also seems likely that the low cost of force in stretch-shorten cycles depends on a specific timing of activation that may not give the required pattern of force to support bodyweight, and requires stretch and shortening to occur in the same muscle rather than being distributed across synergistic muscles (Holt et al., 2014; Curtin et al., 2019; Van der Zee et al., 2019; Bohm et al., 2019, 2021a). Hence, the replacement of contractile element work with SEE stretch and recoil may allow for reduced metabolic cost under a more realistic range of contractile conditions.

The reduction of muscle fiber shortening velocity relative to MTU velocity enabled by ESE storage has also been proposed to reduce the metabolic cost of walking and running (Lichtwark and Wilson, 2006; Gabaldon et al., 2008; Lichtwark and Wilson, 2008; Lichtwark and ´ Barclay, 2010; Bohm et al., 2019; Bohm et al., 2021a; Bohm et al., 2021b; Uchida et al., 2016; Swinnen et al., 2023). For example, in running humans, soleus fibers shorten throughout stance, first stretching the tendon and then continuing to shorten as the tendon recoils. Contractile element shortening velocity is close to that giving maximal efficiency throughout stance, but the MTU shortens more rapidly during the latter half of stance (Bohm et al., 2021b). This reduction in fiber shortening velocity may also reduce cost by allowing for the use of slower, more economical muscle fiber types (Rome et al., 1988; Barclay et al., 1993; Schiaffino and Reggiani, 2011; Labonte and Holt, 2022). Hence, SEEs may save energy not only by recycling the energy of the body, but also by being loaded by muscle work and then recoiling to improve muscle efficiency for a given MTU velocity.

The storage of ESE in SEEs clearly has major and diverse benefits for locomotor performance. These benefits have been usefully characterized as: 1) storing contractile element work in SEEs and then rapidly delivering it to the body to enhance muscle positive power; 2) storing the energy of the body in SEEs and slowly delivering it to the contractile element to attenuate negative muscle power; and 3) storing and returning the energy of the body to reduce muscle work and save metabolic energy (Roberts and Azizi, 2011). However, what has become apparent from the extensive study of these phenomena is the intermediate conditions. For example, the ESE used to enhance power in jumping may arise from the energy of the body rather than from muscle work, the storage and return of ESE can save metabolic energy by allowing for the use of more efficient shortening velocities rather than by eliminating muscle work, and using both the energy of the body and muscle work to store ESE has been suggested to save metabolic energy and enhance power during walking (Fukunaga et al., 2001; Ishikawa et al., 2005). Hence, it may be beneficial to consider both the benefits of stored ESE and the flow of energy, particularly when considering whether MTU design and tuning might be optimized for specific aspects of locomotor performance.

## 5. Task-specific MTU design and tuning

**Fig. 4.**
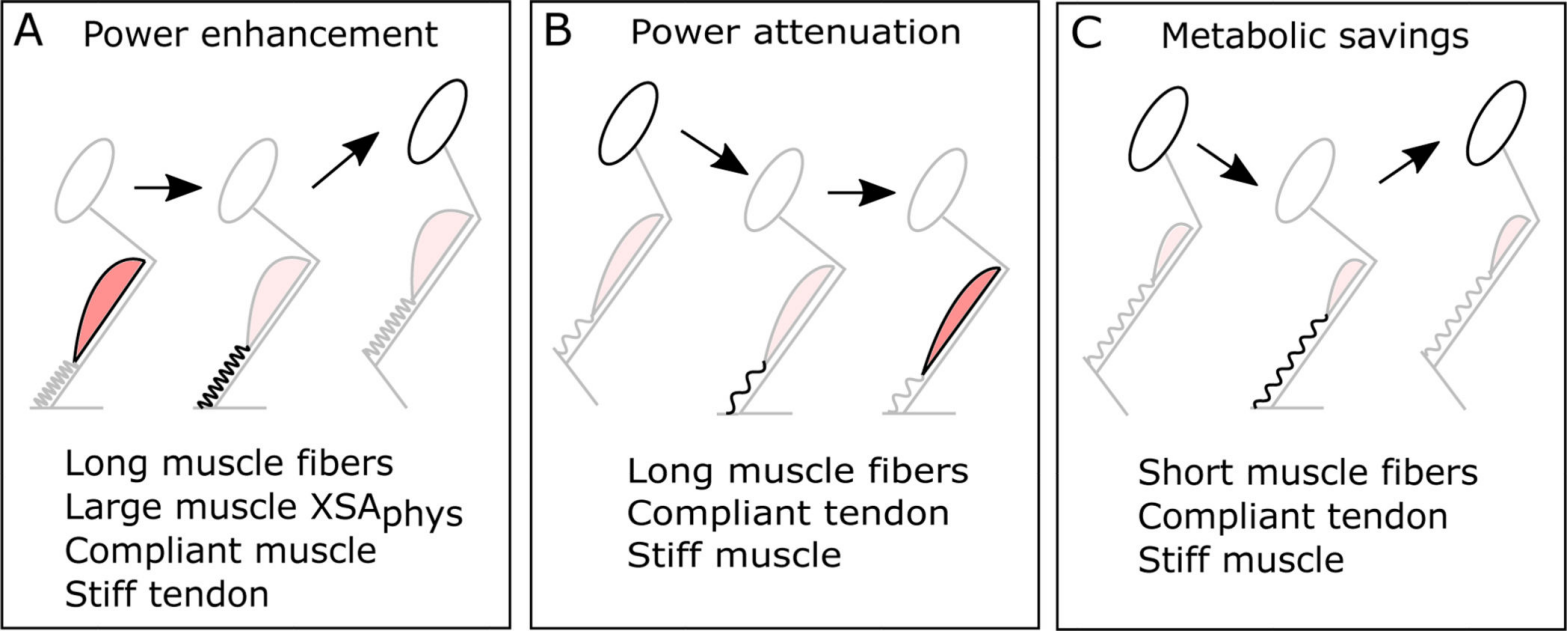
Schematic showing proposed locomotor benefits of ESE storage (adapted from Roberts and Azizi, 2011). The flow of energy between the body, contractile elements, and elastic elements is highlighted in darker colors, and potentially beneficial MTU properties are shown. In power enhancement (A) contractile elements slowly do work on elastic elements, and stored ESE is subsequently delivered to the body rapidly during take-off. In power attenuation (B) the energy of the body stretches the tendon rapidly upon landing and contractile elements generate force to resist stretch. This energy is then delivered to the muscle fibers more slowly and they stretch and dissipate energy. In metabolic energy saving (C), the energy of the body is transferred to SEEs and contractile elements generate force to resist stretch in the first half of stance. This energy is then returned to the body in the second half of stance.

Historically, MTUs have been designated as specialized for ESE storage based on their morphological or functional design (Pollock and Shadwick, 1994b). However, this may be an inadequate distinction given the diversity of ways in which MTUs can store and benefit from ESE. For example, in MTUs where ESE arises from muscle work, and the goal is power enhancement, we might expect to see increased contractile element length to allow for greater active shortening, increased contractile element XSA_phys_ to increase force, stiff SEEs which store more ESE for a given amount of muscle fiber shortening, more compliant muscle fibers which would allow for longer operating lengths and, and tendons that can recoil rapidly (Fig. 4A). While in MTUs where ESE arises predominately from the energy of the body and the goal is power attenuation, we might expect to see long contractile elements that could stretch and dissipate energy, compliant SEEs that would be preferentially stretched as the MTU is actively stretched, stiff muscle fibers that would resist stretch, and SEEs with a high hysteresis that would reduce the energy dissipation required of contractile elements (Fig. 4B). And in MTUs where ESE arises primarily from the energy of the body and the goal is metabolic savings, we might expect to see shorter muscle fibers that would have a lower cost per unit force, stiffer muscle fibers that would resist contractile element stretch, and compliant SEEs that would be preferentially stretched (Fig. 4C). However, we must note that many of these changes are not mutually exclusive and may have detrimental effects if not properly tuned. For example, longer contractile elements will require shorter (and therefore stiffer) SEEs if MTU length is constant, and increased SEE stiffness will limit ESE unless combined with increased contractile element XSA_phys_. Hence, even in the most specialized MTUs we would likely only see some of these effects, and the potential for MTU task-specific design and tuning may be obscured by the need for MTUs to fulfil multiple functions.

There has been little explicit consideration of contractile and elastic element properties in the context of the diversity of ways in which MTUs can store and benefit from ESE. However, we do tend to see the highest fiber length factors in large cursorial mammals thought to be specialized for reduced metabolic cost (Pollock and Shadwick, 1994a), and even the MTUs of highly specialized jumpers do not exhibit fiber length factors low enough to be considered specialized for ESE storage (Ker et al., 1988; Mendoza and Azizi, 2021). This suggests a pattern of relatively longer contractile elements in MTUs specialized for power amplification. We tend to see increased muscle XSA_phys_, increased SEE stiffness (Mendoza and Azizi, 2021) and more compliant muscle fibers (Azizi et al., 2014) in MTUs highly specialized for power amplification, and stiffer muscle fibers in MTUs used in braking (Azizi et al., 2014). It has been speculated that variation in SEE properties (Ilton et al., 2018), such as speed of recoil or hysteresis, might be beneficial. However, no evidence of such adaptation has been observed.

## 6. Conclusions and future directions

Extensive study of contractile and SEEs, the design and tuning of MTUs, and organismal performance has given us great insight into the potential for ESE to be stored in MTUs, and the locomotor benefits of this. However, open questions remain about the role of aponeuroses as SEEs and their variation across MTUs, the metabolic cost of contractile element force and work under the dynamic conditions relevant to locomotion, the more detailed mechanisms of power enhancement and attenuation and metabolic savings, and the potential for MTUs to be specialized for different aspects of locomotor performance rather than simply ESE storage. We suggest that comparative study of aponeurosis as has been undertaken in tendons, detailed muscle energetics studies as have been done for muscle mechanics, and study of MTU design and tuning that explicitly accounts for the source of mechanical energy and aspect of locomotor performance to be enhanced would greatly improve our understanding of the role of SEEs in enhancing muscle and locomotor performance in a comparative context.

## Supplementary Material

supplemental material
