# Supplementary material for: Muscle-tendon unit design and tuning for power enhancement, power attenuation, and reduction of metabolic cost
Source: J Biomech. Author manuscript; Available in PMC 2024 Mar 19. (PMC10949972; doi:10.1016/j.jbiomech.2023.111585)
Supplement: supplemental material [file NIHMS1968210-supplement-supplemental_material.pdf]

Intervention/Comparison	Change in force/torque (%)	Change in stiffness (%)	Reference
Training effect	30	23	(Seynnes et al., 2009)
	12	35	(McMahon et al., 2013)
	19	43	(McMahon et al., 2013)
	24	50	(McMahon et al., 2013)
	24	14	(Massey et al., 2018)
	17	20	(Massey et al., 2018)
	42	57	(Kubo et al., 2001)
	20	51	(Kubo et al., 2006)
	18	10	(Kubo et al., 2006)
	47	46	(Waugh et al., 2018)
	27	51	(Kubo et al., 2012)
	49	82	(Geremia et al., 2018)
	15	19	(Kubo et al., 2007)
	14	30	(Kubo et al., 2007)
	12	17	(Arampatzis et al., 2010)
	44	36	(Arampatzis et al., 2007a)
	8	34	(Quinlan et al., 2021)
	13	41	(Quinlan et al., 2021)
	7	16	(Albracht and Arampatzis, 2013)
	18	25	(Massey et al., 2018)
	58	17	(Massey et al., 2018)
	14	34	(McMahon et al., 2018)
	22	43	(McMahon et al., 2018)
	22	19	(Fletcher et al., 2010)
	15	16	(Werkhausen et al., 2018)
	14	41	(Centner et al., 2019)
	10	36	(Centner et al., 2019)
	14	15	(Kongsgaard et al., 2007)
	5	-9	(Kongsgaard et al., 2007)
	21	31	(Kubo et al., 2002)
	10	31	(Bohm et al., 2021a)
	18	10	(Walker et al., 2020)
	10	-4	(Walker et al., 2020)
	4	-11	(Kubo et al., 2021)
	3	35	(Hirayama et al., 2017)
	15	50	(Malliaras et al., 2013)
	14	35	(Malliaras et al., 2013)
	30	81	(Malliaras et al., 2013)
	41	71	(Kubo et al., 2009)
	17	37	(Kubo et al., 2017)
	6	24	(Fouré et al., 2010)
Immobilization effect	-24	-20	(Couppé et al., 2012)
	-19	-33	(Kubo et al., 2000)
	-23	-29	(Kubo et al., 2004)
	-28	-58	(Reeves et al., 2005)
	-19	-13	(Kubo et al., 2004)
	-22	-29	(de Boer et al., 2007)

	-29	-1	(Boesen et al., 2013)
Training status	39	31	(Couppe et al., 2014)
	2	-26	(Karamanidis and Arampatzis, 2006)
	8	-14	(Karamanidis and Arampatzis, 2006)
	24	11	(Charcharis et al., 2019)
	17	15	(Charcharis et al., 2019)
	-9	-11	(Kubo et al., 2010)
	-23	-6	(Kubo et al., 2010)
	60	70	(Arampatzis et al., 2007b)
	18	23	(Arampatzis et al., 2007b)
	3	-4	(Rosager et al., 2002)
	18	36	(Couppé et al., 2008)
Sex effect	-45	-26	(Carroll et al., 2008)
	-29	-25	(Stenroth et al., 2012)
	-12	-32	(Muraoka et al., 2005)
	-27	-26	(Kubo et al., 2003)
	-39	-46	(Onambélé et al., 2007)
	-35	-45	(McMahon et al., 2018)

Supplementary table –The effect of training, immobilization, training status (e.g., runners vs non-runners, athletes vs non-athletes), and sex on muscle force or torque and SEE stiffness. All values are expressed as a percent change between the groups under comparison. These values are used in Fig. 2C.
